# Multidimensional Comparative Analysis of Bioactive Phenolic Compounds of Honeys of Various Origin

**DOI:** 10.3390/antiox10040530

**Published:** 2021-03-29

**Authors:** Michał Gośliński, Dariusz Nowak, Artur Szwengiel

**Affiliations:** 1 Department of Nutrition and Dietetics, Faculty of Health Sciences, Ludwik Rydygier Collegium Medicum in Bydgoszcz, Nicolaus Copernicus University, 87-100 Toruń, Poland; d.nowak@cm.umk.pl; 2 Institute of Food Technology of Plant Origin, Faculty of Food Science and Nutrition, Poznań University of Life Sciences, 60-637 Poznań, Poland; artur.szwengiel@up.poznan.pl

**Keywords:** honey, antioxidant properties, phenolic acids, flavonoids, cluster analysis

## Abstract

Honey is a natural product which owes its health benefits to its numerous bioactive compounds. The composition of honey is highly diverse and depends on the type of honey and its origin. Antioxidant capacity arises mainly from the total content of polyphenols and their composition. The aim of this study was to perform a multidimensional comparative analysis of phenolic compounds of honeys of various origins. Honeydew, buckwheat, manuka, Malaysian and goldenrod honeys had the highest antioxidant capacity (above 400 mg Trolox equivalents kg^−1^). These honeys were also characterized by the highest total polyphenol content (about 2500 mg gallic acid equivalents (GAE) kg^−1^) and the highest total flavonoid content (1400–1800 mg catechin equivalents (CAE) kg^−1^). Other honeys had much lower antioxidant properties. A multidimensional analysis of the profiles of phenolic compounds showed that honeys constitute a non-homogeneous data set and manuka honey was in contrast to other samples. Principal component analysis (PCA) (based on 18 phenolic compounds) distinguished honeys into five groups. Manuka, Malaysian and honeydew honeys created their own separate groups and the location of other honeys was variable. Ultra-high-performance liquid chromatography (UHPLC) analysis demonstrated that profiles of polyphenols in honeys were highly varied. Caffeic acid, datiscetin and rhamnetin were characteristic compounds for manuka honey. Quercetin, kaempferol and apigenin were present in all honeys except Malaysian honey. The antioxidant properties and the profiles of bioactive phenolic compounds of honeys were miscellaneous. The richest sources of polyphenols were local buckwheat and honeydew honeys, alongside exotic manuka and Malaysian honeys. These honeys could provide valuable ingredients to the human diet, helping to prevent diseases.

## 1. Introduction

Honey has been used by people for ages; in fact, it was used in antiquity, owing to its sweetening and healthful properties [1]. The global production of honey is estimated at about 1.2 million tons; the leaders are China (24%) and the EU (14%) [2,3]. The consumption of honey varies worldwide, ranging from ca. 3 kg/person/year in the USA and ca. 0.9 kg in the EU, with the highest consumption in Greece (3.5 kg), Austria (2.5 kg) and Germany (2 kg). Despite the stable honey supply in Poland, consumption is not high (ca. 0.65 kg), and preferred types are multifloral, linden and acacia [4]. The average price of multifloral honey at the site of production in the EU was 6.46€/100 kg, ranging from 19.25€ in Ireland to 2.25€ in Romania (5.81€ in Poland) [2]. In the last few years exotic honeys have become more popular, e.g., manuka and Malaysian honeys. These are available at health food stores, although unfortunately their prices are very high (ca. 60€/500 g).

The composition of honey is highly varied and depends on the type of honey, the flowers and pollen that bees collect, etc. Honey consists of over 180 substances, but its main components are fructose (38%) and glucose (31%). Other sugars found in honey are disaccharides, such as maltose, sucrose, maltulose, turanose and many others [5,6]. Honey also has small amounts of vitamins (riboflavin, niacin, folic acid, pantothenic acid, vitamin B_6_, ascorbic acid) and minerals (calcium, iron, zinc, potassium, phosphorus, magnesium, selenium, chromium, manganese), as well as organic acids (acetic, butyric, citric, succinic, lactic, malic and gluconic acid) [7]. Its composition also comprises amino acids, proteins, enzymes (glucose oxidase, sucrose diastase, catalase and acid phosphatase) and polyphenols, mostly flavonoids and phenolic acids [1,6,8,9]. The group of flavonoids present in honey encompasses flavonols (myricetin, kaempferol, 8-methoxy kaempferol, quercetin, isorhamnetin, quercetin-3-methyl ether, quercetin 3, 7-dimethyl ether, pinobanksin, rutin and galangin), flavons (genkwanin, luteolin, apigenin, tricetin and chrysin) and flavanones (pinocembrin and pinostrobin). Phenolic acids in honey are represented by hydroxybenzoic acids (methyl syringate, gallic acid, ellagic acid, protocatechuic acid, syringic acid, benzoic acid, 4-hydroxybenzoic acid), hydroxycinnamic acids (chlorogenic, vanillic, caffeic, *p*-coumaric, ferulic acids) and hydroxyphenylacetic acids (homogentisic and phenylacetic acids) [1,6,10,11,12,13]. Research reveals that both flavonoids and phenolic acids endow honey with antioxidant capacity and other medicinal properties [6,14,15]. For instance, honey can prevent oxidative stress, which causes aging and induces many chronic and degenerative diseases, i.e., neoplasms and atherosclerosis [6,16]. It has been demonstrated that the consumption of honey in a dose of 1.2 g kg^−1^ of body weight dissolved in 250 mL water, accompanied by a healthy diet, resulted in a rise of the activity of such antioxidants as β-carotene, vitamin C, glutathione reductase and ureic acid in healthy people [17]. It has been claimed that dark honeys tend to possess greater antioxidant capacity and a higher content of polyphenols than light-color honeys [18,19].

Bioactive compounds present in honeys promote their wide application in the prevention and treatment of many diseases, as well as their use in cosmetology. Various ingredients of honey possess antimicrobial, antioxidant and anti-inflammatory properties [20]. Many authors have shown that honey improved the lipid profile and other factors of cardiovascular diseases [21], and was very effective in the dressing of wounds, burns, skin ulcers and inflammations [22]. Honey-based cosmetics include hydrating creams, tonic, lotions, cleaning milks, shampoos, conditioners and lip ointments. The used amounts usually range between 1% and 10% [23].

The chemical composition and the content of bioactive compounds differentiate honeys and define their pro-health attributes. Therefore, it is necessary to explore these compounds in honeys. Several methods are employed to investigate the structure of phenolic compounds—high-performance liquid chromatography (HPLC), ultra-performance liquid chromatography (UPLC), capillary electrophoresis (CE) and, sporadically, gas chromatography (GC) [1]. We chose an advanced method, UHPLC (ultra-high-performance liquid chromatography), which ensures better resolution and sensitivity. Therefore, the aim of the study was the multidimensional comparative analysis of bioactive phenolic compounds of honeys of various origins.

## 2. Materials and Methods

### 2.1. Samples

The study comprised 21 varied honey samples, each tested in 3 replicates (acacia, buckwheat, goldenrod, honeydew, heather, linden, multifloral, phacelia, rapeseed, raspberry, sunflower and some exotic honeys, i.e., Malaysian and manuka honeys). Artificial honey was used as a control sample. A detailed list of the tested honeys is presented in Table 1. All samples of domestic honeys came from north-eastern Poland (Warmia and Mazury, Podlasie, Kujawy-Pomeranian regions). Exotic honeys (manuka and Malaysian) were imported from New Zealand and Malaysia, respectively. The honeys were purchased from a local health food store. All the honeys were produced and analyzed in 2018. Prior to analysis, the samples were stored at room temperature in the dark. Antioxidant properties were determined in honey solutions. A weighed amount of each honey (1 ± 0.001 g) was diluted in distilled water, in a 1:10 (*w*/*v*) ratio, then homogenized using a vortex mixer for 30 s. For each honey, three parallel samples were prepared.

### 2.2. Antioxidant Capacity by DPPH Assay

The antioxidant capacity of the honey samples was determined by a modified Yen and Chen [24] method, using 0.1 mM methanol solution of a 1,1-diphenyl-2-picrylhydrazyl (DPPH, Sigma-Aldrich). This method is widely used to test the antioxidant capacity of food products, especially fruit, vegetables, juices [25,26,27] and even honeys [28]. A total of 0.1 mL of the sample was added to 2.9 mL of 0.1 mmol L^−1^ DPPH methanol solution (Sigma-Aldrich, Taufkirchen, Germany), mixed and left in the dark for 30 min to incubate at room temperature. The absorbance was measured on a Hitachi U-1900 spectrophotometer (Hitachi, Tokyo, Japan) at 517 nm. For each honey, samples were analyzed in three replicates, from which a mean value was calculated. The percentage of DPPH scavenging was calculated using Equation (1):

% scavenging = [(A_DPPH_ − A_honey_)/A_DPPH_] × 100 (1)

where A_DPPH_ is the absorbance of the DPPH blank solution and A_honey_ is the absorbance of the sample solution.

The value thus achieved was then substituted into an equation of a previously prepared 6-hydroxy-2,5,7,8-tetramethylchromane-2-carboxylic acid (Trolox-Sigma-Aldrich) calibration curve. The antioxidant capacity was expressed as milligrams of Trolox equivalents (Sigma-Aldrich) per kg of a honey sample (mg Tx kg^−1^).

### 2.3. Antioxidant Capacity by ABTS Assay

An antioxidant assay kit (ELISA–Cayman Chemical Company, Ann Arbor, MI, USA) was used to determine the antioxidant capacity of the samples. The assay relies on the ability of antioxidants in a sample to inhibit the oxidation of ABTS (2,2′-Azino-di-[3ethylbenzthiazoline sulphonate]) to ABTS^•+^ by metmyoglobin. The absorbance was measured on a SPEKTROstar Nano (BMG LABTECH) microplate reader at 750 nm after incubation in a shaker for 5 min at room temperature. The antioxidant capacity was quantified as millimolar Trolox equivalents (mM Trolox).

### 2.4. Total Polyphenol Content

The total polyphenol content (TP) of the samples was determined by the Folin–Ciocalteu assay (Sigma-Aldrich) [29]. A total of 0.3 mL aliquot of the sample, 0.05 mL 2N Folin–Ciocalteu reagent, 0.5 mL 20% Na_2_CO_3_ and 4.15 mL distilled water were added to a 10 mL test tube and mixed. The absorbance was measured on a Hitachi U-1900 spectrophotometer at the wavelength λ = 765 nm after 30 min of incubation in the dark at room temperature. The results are expressed as milligrams of gallic acid equivalents per kg of honey sample (mg GAE kg^−1^).

### 2.5. Fast Blue BB Assay

Fast Blue BB is a novel method described by Medina [30] to quantify the phenolic compounds through direct interaction of polyphenols with the Fast Blue BB (FBBB) reagent (4-benzoylamino-2,5-diethoxybenzenediazonium chloride hemi(zinc chloride) salt; Sigma-Aldrich) in an alkaline medium. This method demonstrates higher values of gallic acid equivalents (GAE) than the Folin–Ciocalteu assay does [30,31]. A 0.2 mL aliquot of 0.1% Fast Blue BB reagent was added to 2 mL of samples and mixed for 1 min and 0.2 mL 5% sodium hydroxide was added. The absorbance was measured on a Hitachi U-1900 spectrophotometer at 420 nm after 90 min of incubation in the dark at room temperature. The results are expressed as gallic acid equivalents per kg of honey sample (mg GAE kg^−1^).

### 2.6. Total Flavonoid Content

The total flavonoid content was measured using the colorimetric assay developed by Kapci et al. [32]. Briefly, 0.3 mL of 5% sodium nitrite was added to 1 mL of the sample at zero time. After 5 min, 0.3 mL of 10% aluminium chloride was added. At the 6th min, 2 mL of 1 M sodium hydroxide was added. The mixture was diluted by the addition of 2.4 mL distilled water and mixed. The absorbance was measured on a Hitachi U-1900 spectrophotometer at 510 nm. The total flavonoid content was determined by a (+)-catechin (Sigma) standard curve and results are expressed as milligrams of catechin equivalents per kg of honey sample (mg CAE kg^−1^).

### 2.7. LC-MS Analysis

Reversed-phase (C18 column) ultra-high-performance liquid chromatography electrospray ionization mass spectrometry (RP-UHPLC-ESI-MS) analysis was performed using a Dionex UltiMate 3000 UHPLC (Thermo Fisher Scientific, Sunnyvale, CA, USA) coupled to a Bruker maXis impact ultra-high-resolution orthogonal quadrupole-time-of-flight accelerator (qTOF) equipped with an ESI source and operated in the positive-ion mode (Bruker Daltonik, Bremen, Germany). The reversed phase (RP) chromatographic separation was achieved with a Kinetex™ 1.7 μm C18 100 Å, LC column 100 × 2.1 mm (Phenomenex, Torrance, CA, USA) according to the method of Biesaga and Pyrzyńska [33]. The mobile phase was composed of water containing 1% formic acid (A) and acetonitrile containing 5% water and 1% formic acid (B). The flow rate was 0.2 mL/min with a gradient elution of 5–95% B over 35 min, and standing at 95% B for 20 min. The sample injection volume was 2 μL. The detailed LC-MS settings were previously described by Mildner-Szkudlarz et al. [34] and Nowak et al. [35].

### 2.8. Extraction Procedure

The honey samples were extracted with 40% methanol:60% acidified water (pH 2, HCl). The samples were mixed with liquid in a ratio of 1:5 then stirred in a magnetic stirrer for 15 min [33]. Three independent extraction trials were performed. Samples were centrifuged at 15,000× *g* and then injected to LC-MS for analysis.

### 2.9. Statistical Analysis

The results of antioxidant capacity assays were statistically analyzed by calculating the mean and standard deviation. The interpretation of the results was performed with MS Excel 2010 Analysis Tool-Pak software (Microsoft, Redmond, WS, USA), with one-way analysis of variance (ANOVA) using Tukey’s post-test: different letters in the same row or column indicate statistical significance (at *p* ≤ 0.05).

Chromatographic separations of all samples were carried out in two independent replicates. The bucket table was calculated for all samples (LC-MS data) with Profile Analysis 2.1, using the Find Molecular Features (FMF) algorithm (Bruker Daltonik, Bremen, Germany). Each bucket is represented by a pair of retention times and mass to charge values (*m*/*z*). The bucket intensity values of each LC-MS run were calculated. The values collected in the bucket table were used for statistical analysis. Cluster analysis (CA) and principal component analysis (PCA) were used to compare samples and extract the most discriminating compounds in tested samples. The statistical analysis was performed using Statistica software (Version 12, StatSoft Inc., Tulsa, OK, USA).

## 3. Results and Discussion

First, the antioxidant properties of all the tested honeys were determined (Table 1). The antioxidant capacity was the highest for honeydew 2 (613 ± 20 mg Tx kg^−1^). Slightly lower antioxidant properties were observed in honeydew 1 and buckwheat 2 (542 ± 7 and 511 ± 9 mg Tx kg^−1^, respectively). A relatively high antioxidant capacity was determined in exotic honeys (Malaysian and manuka honeys) and goldenrod (all above 400 mg Tx kg^−1^). The lowest antioxidant capacity, excluding artificial honey, were possessed by acacia, sunflower, linden, multifloral 1 and rapeseed honeys (from 94 ± 1 to 139 ± 3 mg Tx kg^−1^). The highest antioxidant capacity (determined by the ABTS method) was also found in honeydew 1 and honeydew 2, goldenrod and exotic honeys (above 2.0 mM Tx). Buckwheat honeys (samples 1 and 2) had slightly lower antioxidant capacity (1.78 ± 0.10 and 1.83 ± 0.20 mM Tx, respectively). The antioxidant characteristics of the analyzed honeys originated from the presence of numerous bioactive compounds, mainly polyphenols, in their composition. The highest total polyphenols were in buckwheat 2 and 1 honeys (3508 ± 178 and 2468 ± 113 mg GAE kg^−1^, respectively), honeydew 2 and 1 (2648 ± 88 and 2434 ± 62 mg GAE kg^−1^, respectively) and goldenrod and manuka honeys (about 2500 mg GAE kg^−1^). The significantly lowest TP (*p* ≤ 0.05) was determined in acacia and sunflower honeys (763 ± 23 and 824 ± 27 mg GAE kg^−1^, respectively). Similar observations were achieved when total polyphenols were determined with the FBBB method (Table 1). In this case, too, the lowest values were identified in sunflower and acacia honeys (492 ± 12 and 503 ± 11 mg GAE kg^−1^), whereas the highest values were obtained for buckwheat 2, honeydew 2, Malaysian and manuka honeys (above 1800 mg GAE kg^−1^).

Data are a mean ± standard deviation (*n* = 3). Statistical analysis was performed by means of one-way ANOVA using Tukey’s post hoc test—different letters in the same column indicate statistical significance (at least *p* ≤ 0.05). Abbreviations: DPPH, 1,1-diphenyl-2-picrylhydrazyl; ABTS-2,2′-azino-di-[3ethylbenzthiazoline sulphonate]; Tx, Trolox equivalents; TP, total polyphenol content; GAE, gallic acid equivalents; TF, total flavonoid content; CAE, catechin equivalents.

In the study reported herein, the content of total polyphenols ranged from ca. 800 to 3500 mg GAE kg^−1^. These results were approximately the same as those demonstrated by Al-Mamary et al. [36], who determined TP in different types of diluted honey with the Folin–Ciocalteu assay and achieved TP from 563.2 to 2462.1 mg GAE kg^−1^, but converted them to catechin equivalents. Attanzio et al. [37] applied the Folin–Ciocalteu method to determine total polyphenols in 33 types of honey, and the values obtained varied from 165 to 1333 mg GAE kg^−1^. The total polyphenol content differed depending on the plant family; for example, the lowest value was in honeys from *Leguminosae* (i.e., acacia, carob) and the highest was found in *Apiaceae* (ferula, dill) [37]. Lower TP values were obtained by Moniruzzaman et al. [38]. The total phenolic content of the honeys tested in their study was between 144.5 and 580.0 mg GAE per kg of honey [38]. In our investigations, the highest total polyphenol content was observed in buckwheat honeys. The same type of honeys was found to contain the highest TP in a study by Gheldof and Engeseth [39], but the values they reported were several times lower (ca. 800 mg GAE kg^−1^) than those in our study. We noticed that honeydew honeys had two- to three-fold higher total polyphenols than nectar honeys. A similar conclusion was drawn by Attanzio et al. [37]. We determined a relatively high content of TP in manuka honeys (over 2400 mg GAE kg^−1^). Manuka honey had the highest total polyphenols in a study carried out by Alzahrani et al. [40], but the values obtained by those researchers were more than 2.5-fold lower than those achieved in our tests (899.1 ± 11.8 mg GAE kg^−1^). Moniruzzaman et al. [38] determined an even lower total amount of polypehnols in manuka honey, i.e., 429.6 mg GAE kg^−1^. In our study, manuka honeys had a slightly higher total polyphenol content than Malaysian honey. Khalil et al. [41] reported that manuka honey had as much as two- to three-fold higher TP than Malaysian honeys of different origins. On the other hand, Moniruzzaman et al. [38] observed that some Malaysian honeys (Longan and Sourwood) had a slightly higher TP content than manuka honey did. Higher values of total polyphenols were determined with the FBBB method than those determined according to Folin–Ciocalteu in some earlier research [30,31]. These observations mostly pertained to juices. In our study, the results of total polyphenols in honeys determined using the FBBB method were much lower than the ones achieved using the Folin–Ciocalteu assay. This could be due to the presence of a large amount of glucose and fructose in honeys. Kang et al. [42] reported that the Folin–Ciocalteu method has been criticized for its lack of specificity, since several non-phenolic substances, e.g., monosaccharides, can interfere with the measurements of total phenols.

Total flavonoid content (TF), as determined in our study, ranged from 402 ± 23 mg CAE kg^−1^ (sunflower) to 1874 ± 148 (honeydew 2) (Table 1). The highest TF values were detected in honeydew 1 and 2, buckwheat and Malaysian honey (above 1700 mg CAE kg^−1^), which were also characterized by a high antioxidant capacity. In the above honeys, TF values corresponded to about 50%–80% of total polyphenols. Similar observations were reported by Khalil et al. [41], who found that the content of flavonoids in the Malaysian honey equaled 60–80% of TP. On the other hand, Attanzio et al. [37] reported a content of flavonoids within a range of 40 to 821 mg kg^−1^, but expressed this as quercetin equivalents. These amounts corresponded to around 25% to 60% of total polyphenols [37]. Furthermore, Moniruzzaman et al. [38] determined much lower values of TF in analyzed honeys, i.e., from 14.2 to 156.8 mg CAE kg^−1^, which corresponded to around 10% to 30% of total polyphenols. High values of TP and TF in the honeys we analyzed, especially in honeydew, buckwheat, goldenrod and exotic honeys (manuka, Malaysian), were reflected by the high antioxidant capacity values of these honeys (determined using both DPPH and ABTS methods). In another study, among seven types of Polish honeys (lime, nectar-honeydew, rape, honeydew, acacia, buckwheat and multiflower), the highest antioxidant capacity (identified using DPPH and ABTS), as well as the highest total polyphenols, were revealed in buckwheat honey [43]. Likewise, Kuś et al. [44] concluded that buckwheat honey was characterized by the highest total polyphenols and antioxidant capacity (DPPH) among the six types of honeys they tested. There are other investigations indicating that honeys with a high content of polyphenols and flavonoids were distinguished by high antioxidant capacity [37,41].

Furthermore, correlation coefficients were calculated, showing a high linear correlation between antioxidant capacity and total polyphenol content, and total flavonoids (0.902 and 0.938, respectively). In addition, a relatively high correlation coefficient between TP and TF (0.893) was determined. Moniruzzaman et al. [38] also demonstrated a high correlation between TP and TF (0.958), although it was slightly lower than the correlation calculated in our study between DPPH and TP (0.789). It should be stated that the antioxidant capacity of honeys mostly depends on polyphenols, and flavonoids play a significant role.

**Figure 1:**
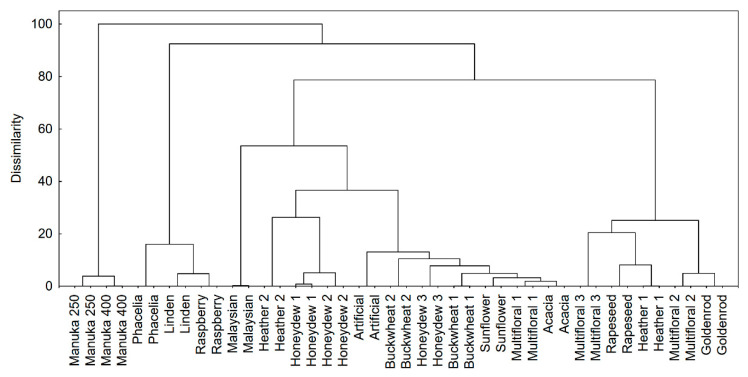
Cluster analysis results—the data set with 1252 compounds was included as a fingerprint of a sample; Ward’s method was used as an amalgamation rule and squared Euclidean distances were used as a distance measure.

In the next stage of the research, multivariate exploratory techniques were used to distinguish variations in the LC-MS profiles of honeys. A cluster analysis was applied to organize the honeys into meaningful structures. A total of 1252 compounds (S/N > 15) were identified in 21 samples of honey using the FMF algorithm. Cluster analysis is an exploratory data analysis tool which aims to sort different objects into groups in such a way that the degree of association between two objects is maximal if they belong to the same group and minimal otherwise. This analysis was used to discover structures in the data without providing any explanation or reasons. The cluster analysis results showed that samples created a non-homogeneous data set, in addition to which manuka honey samples were totally different (Figure 1). Manuka honey was in contrast to other samples. Not all samples of multifloral and heather honeys were in the same clusters. It is obvious that multifloral honey is a heterogenous sample. However, the location of heather 2 samples next to honeydew samples is not surprising, because both samples were dark honey. The chemical profile of the control sample (artificial honey) was close to that of natural honeys. Presumably, flavors added to the artificial honey are typical for natural products because producers offer natural and synthetic flavors and fragrances.

**Figure 2:**
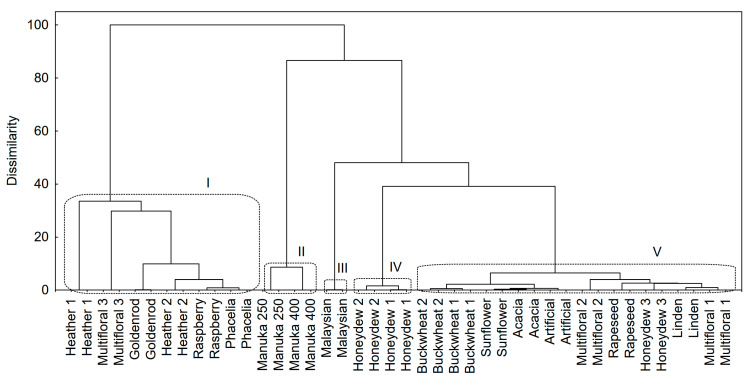
Cluster analysis results—the data set with 18 polyphenolic compounds was included as a bioactive fingerprint of a sample; Ward’s method was used as an amalgamation rule and squared Euclidean distances were used as a distance measure. I–V, cluster numbers.

Some bioactive polyphenolic compounds (phenolic acids and flavonoids) were detected in honey samples (Table 2 and Table 3). If samples were compared according to the variation of polyphenolic compounds (Figure 2), five groups became distinguishable (cutting the dendrogram at 40%). The samples from the 1st group contained a high concentration of polyphenols but these were much lower than those found in manuka honeys (2nd group), which mainly resulted from the content of caffeic acid (Table 2). The samples from the 3rd and 4th groups had similar concentrations of polyphenols but their profiles were totally different. The 5th group was very wide and also included the control sample, although polyphenols were not detected in artificial honey.

Our analysis of the structure of polyphenolic compounds (Table 2 and Table 3) demonstrated that manuka honeys were characterized by a very high content of caffeic acid (from 35.69 ± 0.12 to 38.67 ± 2.02 mg kg^−1^). Manuka honeys, like Malaysian honey, also had the highest content of chlorogenic acid (from 2.68 ± 0.03 to 3.29 ± 0.15 mg kg^−1^) in comparison with the other honeys. It is worth noting that heather 2 and multifloral 3 honeys had the highest content of *p*-coumaric acid (4.09 ± 0.24 and 4.05 ± 0.06 mg kg^−1^, respectively). Slightly less of this compound was detected in goldenrod honey (3.36 ± 0.18 mg kg^−1^). In turn, syringic acid was prevalent in multifloral 3 and manuka honeys. Multifloral 3 honey also comprised the highest content of quercetin (2.08 ± 0.05 mg kg^−1^), whereas manuka honeys had the highest amounts of datiscetin (from 2.09 ± 0.04 to 2.75 ± 0.03 mg kg^−1^). A complete list of phenolic compounds detected in the analyzed honeys is presented in Table 2 and Table 3.

**Figure 3:**
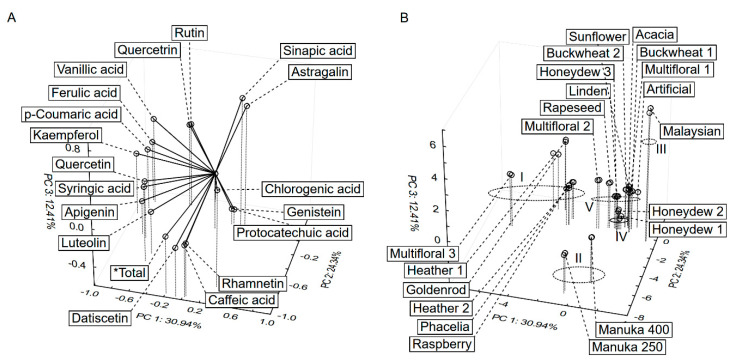
Principal component analysis (PCA) of phenolic profiles of honey samples, loading (**A**) and score plot (**B**).

The PCA was computed for honey samples according to their polyphenolic profiles to see relation between variables and samples. The three PCs explained 68% of the total variance (Figure 3). The five groups were circled (Figure 3B) analogously to the cluster analysis results presented above. The samples from the 1st group (heather, phacelia, goldenrod) contain most of the detected polyphenols. The position of manuka samples (2nd group) was determined by their high concentration of caffeic acid, datiscetin and rhamnetin. Sinapic acid and astragalin dominated in the samples from Malaysia (3rd). Genistein was detected only in samples from the 4th group (honeydew), and these samples had relatively high concentrations of protocatechuic acid. The 5th group contained a lot of honey samples, of which the location was determined by chlorogenic acid.

In light of the current research results, high-performance and ultra-performance liquid chromatography (HPLC and UPLC) are very accurate and sensitive methods for the analysis of polyphenolic compounds [1,45]. In our study, we determined the content of 18 phenolic compounds, and the dominant one was caffeic acid. Subsequently, in smaller quantities we observed *p*-coumaric acid and chlorogenic acid, as well as quercetin and datiscetin. Most caffeic acid was detected in manuka honeys, whereas *p*-coumaric acid was most abundant in heather 2 and multifloral 3 honeys. Caffeic acid had been previously determined in many honeys, e.g., Australian eucalyptus honeys and manuka honey (from Australia and New Zealand) as well as acacia, heather, chestnut, lavender, rosemary, orange, sunflower and rapeseed honeys. These honeys also contained *p*-coumaric acid [1]. In another study conducted by Marshall et al. [46], the identification of phytochemicals in honeys (20 honey samples, 15 monofloral and 5 multifloral honeys from Florida) by HPLC-DAD-ESI-MS revealed the presence in the analyzed honeys of 10 phenolic compounds (coumaric acids, rutin, 2-trans,4-trans-abscisic acid, 2-cis,4-trans-abscisic acid, quercetin, luteolin, kaempferol, pinocembrin and chrysin + galangin and two unidentified compounds). Most *p*-coumaric acid was identified in multifloral honey (2.39 ± 0.33 mg kg^−1^), but this compound was absent in manuka honeys [46]. We detected *p*-coumaric acid in manuka honey, although it was present in a low concentration. *p*-coumaric acid was the leading compound in four of the seven analyzed samples of buckwheat honeys (34.56–45.51 mg kg^−1^), next to 4-hydroxybenzoic acid [47]. Very small quantities of *p*-coumaric acid were detected in the other three samples of buckwheat honeys, which confirms the fact that the composition of phenolic acids is diverse and differences can appear even in the same type of honey. Socha et al. [43] analyzed seven types of honey and detected the highest content of phenolic acids in buckwheat honey, followed by linden and honeydew honeys. The dominant phenolic acids in buckwheat honey were gallic and caffeic acids (9.13 and 7.07 mg kg^−1^, respectively). Acacia honey contained the least phenolic acids, both in our study and in the research reported by Socha et al. [43]. On the other hand, Zhang et al. [48] showed that acacia honey had a considerable content of gallic acid (c.a. 4.4 mg kg^−1^), and similar amounts of other phenolic acids as in the other honeys they analyzed, i.e., milk vetch, wild chrysanthemum and jujube honeys.

Another important group of phenolic compounds in honey are flavonoids [1,6,10]. In our research, all honeys except Malaysian honey were found to contain kaempferol (from 0.11 to 2.27 mg kg^−1^). In a study by Petrus et al. [49], most of this compound was determined in melon, pumpkin and rapeseed honeys (about 3 mg kg^−1^). Kaempferol was also identified in many samples of buckwheat and heather honeys (from 0.10 to 3.74 mg kg^−1^) [47], as well as in tupelo, citrus and multifloral honeys (from 0.62 to 3.12 mg kg^−1^) [46]. Rutin was not detected neither in our honeys (except heather 1), nor was it detected by Marshall et al. [46]. Quercetin was detected within a wide range of 0.11 to 2.08 mg kg^−1^ (the highest quantity in multifloral 3) in most of the honeys we analyzed. In another study, quercetin was present in most of the honeys, within a range of 0.31 to 1.22 mg kg^−1^ (mostly in tupelo and citrus honey) [46].

## 4. Conclusions

Buckwheat and honeydew honeys, together with manuka honeys, had the highest levels of total polyphenols and the highest antioxidant capacity. This resulted from the presence of flavonoids and phenolic acids. The FBBB method yielded lower values of total polyphenols than the Folin–Ciocalteu assay did, which is not concordant with the results of previous studies conducted on juices. Therefore, further determinations are needed before this method can be recommended for use on honeys. RP-UHPLC-ESI-MS analysis allowed us to identify the presence of 1252 compounds in the analyzed honeys, and this finding led to the performance of a cluster analysis. The outcome showed that samples created a non-homogeneous data set, and manuka honey was in contrast to other samples. The tested honeys were determined on the basis of 18 polyphenolic compounds, and the results served to generate a three-dimensional plot representing the PCA for these compounds. It was possible to distinguish five groups of honeys. Manuka, Malaysian and honeydew honeys were designated into their own separate groups and the location of the other honeys was variable. Analysis based on UHPLC demonstrated that the profiles of phenolic compounds in honeys were diverse and depended on the origin of the honey. Manuka honey was rich in caffeic acid, and datiscetin and rhamnetin were additional characteristic compounds for this honey. Malaysian and manuka honeys contained the most chlorogenic acid among all the analyzed honeys. Regarding flavonoids, quercetin, kaempferol and apigenin were present in all the honeys except Malaysian honey. The smallest quantities of phenolic compounds were determined in acacia honey.

## Figures and Tables

**Table 1:** Antioxidant properties of the analyzed honeys. Abbreviations: TP, total polyphenol content; FBBB, Fast Blue BB reagent; TF, total flavonoid content.

Samples	DPPH mg Tx kg^−1^	ABTS mM Tx	TP mg GAE kg^−1^	FBBB mg GAE kg^−1^	TF mg CAE kg^−1^
Acacia	94 ± 1 ^i^	1.40 ± 0.23 ^e^	763 ± 23 ^h^	503 ± 11 ^i^	408 ± 12 ^f^
Artificial	0 ± 0 ^j^	0.20 ± 0.10 ^g^	977 ± 80 ^g^	815 ± 8 ^f^	0 ± 0 ^g^
Buckwheat 1	376 ± 5 ^d^	1.78 ± 0.10 ^c^	2468 ± 113 ^b^	1276 ± 9 ^d^	1047 ± 23 ^c^
Buckwheat 2	511 ± 9 ^b^	1.83 ± 0.20 ^c^	3508 ± 178 ^a^	1824 ± 16 ^a^	1723 ± 98 ^a^
Goldenrod	449 ± 9 ^c^	2.02 ± 0.08 ^b^	2500 ± 127 ^b^	1466 ± 38 ^c^	1416 ± 105 ^b^
Heather 1	211 ± 3 ^f^	1.56 ± 0.19 ^d^	1526 ± 132 ^e^	1279 ± 27 ^d^	794 ± 62 ^d^
Heather 2	271 ± 3 ^e^	1.64 ± 0.15 ^c,d^	2419 ± 173 ^b^	1299 ± 10 ^d^	1517 ± 87 ^b^
Honeydew 1	542 ± 7 ^b^	2.08 ± 0.13 ^b^	2434 ± 62 ^b^	1542 ± 11 ^b, c^	1772± 202 ^a^
Honeydew 2	613 ±20 ^a^	2.41 ± 0.43 ^a^	2648 ± 88 ^b^	1873 ±23 ^a^	1874 ± 148 ^a^
Honeydew 3	265 ± 5 ^e^	1.43 ± 0.12 ^e^	1614 ± 124 ^e^	1014 ± 16 ^e^	604 ± 29 ^e^
Linden	130 ± 3 ^h^	1.09 ± 0.09 ^f^	913 ± 54 ^g^	694 ± 8 ^g^	420 ± 24 ^f^
Malaysian	410 ± 10 ^c,d^	2.11 ± 0.38 ^a b^	2186 ± 199 ^c^	1824 ± 26 ^a^	1761 ± 115 ^a^
Manuka 250	396 ± 6 ^d^	2.33 ± 0.32 ^a^	2493 ± 95 ^b^	1854 ± 7 ^a^	1498 ± 41 ^b^
Manuka 400	450 ± 15 ^c^	2.35 ± 0.67 ^a^	2418 ± 156 ^b^	1623 ± 37 ^b^	1268 ± 65 ^b,c^
Multifloral 1	123 ± 2 ^h^	1.40 ± 0.14 ^e^	943 ± 60 ^g^	600 ± 16 ^h^	408 ± 15 ^f^
Multifloral 2	155 ± 3 ^g^	1.59 ± 0.21 ^d^	1173 ± 132 ^f^	745 ± 18 ^g^	438 ± 30 ^f^
Multifloral 3	364 ± 12 ^d^	2.00 ± 0.37 ^b^	1848 ± 69 ^d^	1299 ± 41 ^d^	1373 ± 11 ^b^
Phacelia	238 ± 5 ^e,f^	1.57 ± 0.02 ^d^	1872 ± 94 ^d^	918 ± 27 ^e^	715 ± 53 ^d^
Rapeseed	139 ± 3 ^g,h^	1.41 ± 0.07 ^e^	1016 ± 162 ^f,g^	820 ± 4 ^f^	494 ± 31 ^e^
Raspberry	162 ± 3 ^g^	1.67 ± 0.11 ^c, d^	1091 ± 59 ^f^	699 ± 24 ^g^	568 ± 62 ^e^
Sunflower	123 ± 2 ^h^	0.97 ± 0.06 ^f^	824 ± 27 ^h^	492 ± 12 ^i^	402 ± 23 ^f^

Data are a mean ± standard deviation (*n* = 3). Statistical analysis was performed by one-way ANOVA using the Tukey’s *post hoc* test: different letters in the same column indicate statistical significance (at least *p* ≤ 0.05).

**Table 2:** Phenolic compounds of honeys—phenolic acids [mg kg^−1^].

h	Caffeic Acid	Chlorogenic Acid	Ferulic Acid	*p*-Coumaric Acid	Protocatechuic Acid *	Sinapic Acid	Syringic Acid *	Vanillic Acid
Acacia	nd	nd	0.25 ± 0.02 ^e^	nd	nd	nd	nd	0.18 ± 0.01 ^c^
Phacelia	0.45 ± 0.02 ^d^	0.88 ± 0.03 ^d^	1.18 ± 0.03 ^b^	2.83 ± 0.11 ^b^	nd	nd	0.12 ± 0.01 ^e^	0.54 ± 0.02 ^b^
Buckwheat 1	nd	0.69 ± 0.03 ^d^	nd	0.83 ± 0.05 ^e^	nd	nd	nd	nd
Buckwheat 2	nd	0.56 ± 0.03 ^e^	nd	2.13 ± 0.09 ^c^	nd	nd	nd	nd
Linden	0.69 ± 0.04 ^c^	0.73 ± 0.01 ^d^	0.21 ± 0.01 ^e^	nd	nd	nd	0.11 ± 0.01 ^e^	nd
Malaysian	0.65 ± 0.09 ^c^	3.06 ± 0.13 ^a^	0.11 ± 0.01 ^f^	0.46 ± 0.01 ^g^	nd	0.07 ± 0.01 ^a^	0.22 ± 0.01 ^d^	0.33 ± 0.02 ^b,c^
Raspberry	0.44 ± 0.03 ^d^	0.29 ± 0.02 ^f^	1.55 ± 0.06 ^a^	2.44 ± 0.03 ^c^	nd	nd	0.17 ± 0.01 ^d^	0.52 ± 0.03 ^b^
Manuka 250	38.67 ± 2.02 ^a^	2.68 ± 0.03 ^b^	nd	0.84 ± 0.04 ^e^	nd	nd	0.64 ± 0.05 ^b^	nd
Manuka 400	35.69 ± 0.12 ^a^	3.29 ± 0.15 ^a^	nd	0.64 ± 0.04 ^f^	nd	nd	0.4 ± 0.02 ^c^	nd
Goldenrod	0.53 ± 0.04 ^c,d^	nd	1.66 ± 0.04 ^a^	3.36 ± 0.18 ^b^	nd	nd	0.21 ± 0.02 ^d^	1.05 ± 0.04 ^a^
Rapeseed	nd	0.56 ± 0.05 ^e^	0.32 ± 0.03 ^d,e^	0.65 ± 0.02 ^f^	nd	nd	0.22 ± 0.02 ^d^	nd
Sunflower	nd	0.49 ± 0.04 ^e^	0.14 ± 0.01 ^f^	nd	nd	nd	nd	0.20 ± 0.01 ^c^
Honeydew 3	0.59 ± 0.02 ^c^	0.74 ± 0.05 ^d^	0.44 ± 0.05 ^d^	1.45 ± 0.08 ^d^	0.29 ± 0.02 ^b^	nd	0.13 ± 0.02 ^e^	nd
Honeydew 1	1.2 ± 0.03 ^b^	0.78 ± 0.1 ^d^	0.31 ± 0.02 ^d,e^	0.95 ± 0.02 ^e^	0.66 ± 0.03 ^a^	nd	nd	nd
Honeydew 2	0.92 ± 0.07 ^b^	0.83 ± 0.05 ^d^	0.22 ± 0.01 ^e^	0.63 ± 0.05 ^f^	0.77 ± 0.01 ^a^	nd	0.11 ± 0.02 ^e^	0.27 ± 0.01 ^c^
Artificial	nd	nd	nd	nd	nd	nd	nd	nd
Multifloral 1	nd	1.34 ± 0.04 ^c^	nd	nd	nd	nd	nd	nd
Multifloral 2	1.09 ± 0.01 ^b^	1.35 ± 0.08 ^c^	0.38 ± 0.04 ^d^	1.59 ± 0.07 ^d^	nd	nd	0.2 ± 0.01 ^d^	0.45 ± 0.03 ^b^
Multifloral 3	nd	0.36 ± 0.01 ^f^	1.31 ± 0.09 ^b^	4.05 ± 0.06 ^a^	nd	nd	1.05 ± 0.03 ^a^	0.97 ± 0.04 ^a^
Heather 1	nd	0.46 ± 0.04 ^e^	0.71 ± 0.02 ^c^	2.21 ± 0.06 ^c^	nd	nd	0.16 ± 0.02 ^d^	0.55 ± 0.03 ^b^
Heather 2	0.4 ± 0.05 ^d^	nd	1.10 ± 0.01 ^b^	4.09 ± 0.24 ^a^	nd	nd	nd	0.43 ± 0.02 ^b^

* calculated as ferulic acid; nd—not detected; data are means ± standard deviation (*n* = 3); statistical analysis was performed by one-way ANOVA using Tukey’s post hoc test: different letters in the same column indicate statistical significance (at least *p* ≤ 0.05).

**Table 3:** Phenolic compounds of honeys—flavonoids [mg kg^−1^].

Sample	Apigenin	Astragalin **	Datiscetin **	Genistein **	Kaempferol **	Luteolin **	Quercetin	Quercetrin **	Rhamnetin **	Rutin **
Acacia	0.04 ± 0 ^d^	nd	nd	nd	0.15 ± 0.01 ^g^	nd	nd	nd	nd	nd
Artificial	nd	nd	nd	nd	nd	nd	nd	nd	nd	nd
Buckwheat 1	0.03 ± 0 ^d^	nd	nd	nd	0.08 ± 0.01 ^g^	nd	nd	nd	nd	nd
Buckwheat 2	0.03 ± 0 ^d^	nd	nd	nd	0.11 ± 0 ^g^	nd	0.11 ± 0 ^f^	nd	nd	nd
Goldenrod	0.16 ± 0.02 ^b^	nd	nd	nd	1.18 ± 0.05 ^c^	0.06 ± 0 ^d^	0.31 ± 0.01 ^e^	0.04 ± 0.01 ^b^	nd	0.07 ± 0 ^b^
Heather 1	0.06 ± 0.01 ^d^	nd	nd	nd	1.42 ± 0.02 ^b^	0.06 ± 0.01 ^d^	0.94 ± 0.03 ^b^	0.11 ± 0 ^a^	nd	0.15 ± 0.01 ^a^
Heather 2	0.26 ± 0 ^a^	0.07 ± 0 ^c^	0.07 ± 0.01 ^e^	nd	0.97 ± 0.02 ^c^	0.08 ± 0 ^d^	0.21 ± 0 ^e^	nd	nd	nd
Honeydew 1	0.11 ± 0.01 ^c^	0.12 ± 0.01 ^b^	0.23 ± 0.03 ^c,d^	0.41 ± 0.03 ^a^	0.47 ± 0.04 ^e^	0.04 ± 0 ^d^	0.54 ± 0.05 ^c^	nd	nd	nd
Honeydew 2	0.09 ± 0 ^c^	0.11 ± 0 ^b^	0.16 ± 0 ^d^	0.28 ± 0.03 ^b^	0.43 ± 0.04 ^e^	0.04 ± 0 ^d^	0.44 ± 0.1 ^c,d^	nd	nd	nd
Honeydew 3	0.07 ± 0 ^c,d^	nd	nd	nd	0.33 ± 0 ^e,f^	0.03 ± 0 ^d^	0.25 ± 0.01 ^e^	nd	nd	nd
Linden	0.09 ± 0.01 ^c^	nd	0.09 ± 0 ^e^	nd	0.38 ± 0 ^e^	0.07 ± 0 ^d^	0.21 ± 0 ^e^	nd	nd	nd
Malaysian	nd	0.66 ± 0 ^a^	0.06 ± 0 ^e^	nd	nd	nd	nd	nd	nd	nd
Manuka 250	0.15 ± 0 ^b^	nd	2.75 ± 0.03 ^a^	nd	0.43 ± 0.03 ^e^	0.3 ± 0 ^a^	0.64 ± 0 ^c^	nd	0.21 ± 0.01 ^a^	nd
Manuka 400	0.12 ± 0 ^c^	nd	2.09 ± 0.04 ^b^	nd	0.46 ± 0.01 ^e^	nd	0.64 ± 0.02 ^c^	nd	0.24 ± 0 ^a^	nd
Multifloral 1	0.06 ± 0.01 ^d^	nd	nd	nd	0.29 ± 0.03 ^f^	nd	0.14 ± 0 ^f^	nd	nd	nd
Multifloral 2	0.12 ± 0.01 ^c^	0.05 ± 0 ^c^	nd	nd	0.84 ± 0.05 ^c,d^	nd	0.42 ± 0 ^d^	nd	nd	nd
Multifloral 3	0.2 ± 0 ^b^	nd	0.34 ± 0.02 ^c^	nd	2.27 ± 0.02 ^a^	0.14 ± 0.01 ^c^	2.08 ± 0.05 ^a^	nd	nd	nd
Phacelia	0.19 ± 0 ^b^	nd	0.16 ± 0 ^d^	nd	1.13 ± 0.04 ^c^	0.17 ± 0 ^b,c^	0.37 ± 0.02 ^d^	nd	nd	nd
Rapeseed	0.06 ± 0 ^d^	0.06 ± 0 ^c^	0.08 ± 0 ^e^	nd	0.78 ± 0.02 ^d^	0.04 ± 0 ^d^	0.28 ± 0.03 ^e^	nd	nd	0.05 ± 0 ^b^
Raspberry	0.17 ± 0.01 ^b^	nd	0.45 ± 0.03 ^c^	nd	0.95 ± 0.05 ^c^	0.21 ± 0.01 ^b^	0.23 ± 0.01 ^e^	nd	nd	nd
Sunflower	0.03 ± 0 ^d^	0.05 ± 0 ^c^	nd	nd	0.12 ± 0.01 ^g^	nd	0.23 ± 0.02 ^e^	nd	nd	nd

** calculated as quercetin; nd—not detected; data are means ± standard deviation (*n* = 3); statistical analysis was performed by one-way ANOVA using Tukey’s post hoc test: different letters in the same column indicate statistical significance (at least *p* ≤ 0.05).

## Data Availability

The data presented in this study are available on request from the corresponding author.
